# Total *Panax notoginseng* Saponins Repair the Epidermal Barrier by Regulating a Multi-Pathway Network: Insights from an Integrative RHE Model and Multi-Omics Study

**DOI:** 10.3390/ijms262411775

**Published:** 2025-12-05

**Authors:** Agui Xie, Jianxin Wu, Qing Huang

**Affiliations:** Skin Health and Cosmetic Development & Evaluation Laboratory, China Pharmaceutical University, Nanjing 211198, China; xieagui@stu.cpu.edu.cn

**Keywords:** total *Panax notoginseng* saponins, epidermal barrier, barrier repair mechanism, multi-omics, reconstructed human epidermis model

## Abstract

The abnormal barrier function of the stratum corneum is a significant characteristic of surface-active agent-induced inflammatory skin diseases, and its cause is closely related to the abnormal lipid components of the stratum corneum. Total saponins of *Panax notoginseng* (TSPN) have significant potential in improving inflammatory skin barrier function. This study aims to investigate the barrier repair efficacy of TSPN using the EpiKutis^®^ skin model and to explore the potential mechanisms through multi-omics analysis based on transcriptomics, proteomics, and lipid metabolomics. We found that TSPN could ameliorate Sodium dodecyl sulfate (SDS)-induced barrier impairment in the EpiKutis^®^ model, alleviating stratum corneum thickening and upregulating the expression of barrier-related proteins, e.g., Filaggrin, Involucrin, and Loricrin. Through an integrated multi-omics network, we identified seven key target proteins and screened six lipid metabolites, which are involved in lipid metabolism and exert barrier-repairing effects through five pathways. The result indicated that TSPN might repair the epidermal barrier by regulating the phosphatidylinositol 3 kinase (PI3K)-protein kinase B (AKT)-mediated proliferation pathway, Mitogen-activated protein kinase (MAPK)-mediated apoptotic pathways, sphingolipid synthesis, Calcium/calmodulin-dependent protein kinase II beta (CAMK2B)-mediated actin cytoskeleton regulation, and Inositol-trisphosphate 3-kinase B (ITPKB)-mediated phosphatidylinositol signaling system. Further study is needed to explore the mechanism of the molecular link between lipid abnormalities and skin barrier function.

## 1. Introduction

As the body’s first line of defense, the skin plays a crucial role in barrier function. The skin barrier can be divided into physical, chemical, immune, and microbial barriers. Among these, the physical barrier composed of the stratum corneum “brick wall structure” is the foundation for maintaining skin barrier function [1]. The stratum corneum barrier is composed of enucleated corneocytes and intercellular lipids that are rich in ceramide, cholesterol, and free fatty acids. This lipid matrix is crucial for the lipid skin barrier function. Enucleated corneocytes are surrounded by a cornified envelope, which consists of a densely cross-linked layer of proteins such as filaggrin, loricrin, and involucrin. The cornified envelope, together with the lipid envelope, provides a hydrophobic barrier and minimizes the uptake of most substances into the corneocytes and prevents water loss [2]. Notably, stratum corneum barrier dysfunction is a common determinant that threatens skin health and causes a variety of skin diseases [3]. An abnormal stratum corneum barrier is a common feature of the inflammatory skin diseases psoriasis and atopic dermatitis, as well as in surfactant-induced contact dermatitis, along with other features such as abnormal lipid metabolism, epidermal hyperplasia, stratum corneum thickening, and decreased barrier function [4]. Alterations in lipid composition are considered to be the main cause of skin barrier dysfunction, but the mechanisms causing abnormal lipid composition and the molecular link between lipid abnormalities and skin barrier function are still unclear.

The topical application of active ingredients of Traditional Chinese Medicines (TCMs) in the treatment of skin diseases has unique advantages in that they are multi-component, multi-target, and multi-pathway [5]. *Panax notoginseng* (Burkill) F. H. Chen is the dried root of plants in the genus Panax of the family Araliaceae, and has been identified to contain a variety of chemical components, such as saponins, flavonoids, polysaccharides, amino acids, and fatty acids [6]. Among these, the major bioactive components are the saponins of *Panax notoginseng* that are a kind of chemical mixture containing different dammarane-type saponins, mainly including ginsenosides Rg1, Re, and Rb1 and notoginsenoside R1, which have demonstrated significant potential in treating inflammatory skin diseases due to their various properties, such as removing stasis, reducing bleeding and swelling, and alleviating pain [7]. Studies have shown that the saponins of *Panax notoginseng* can reduce skin thickening, desquamation, and erythema induced by imiquimod in psoriasis mice by reducing skin inflammation [8]. The Wnt/β-catenin signaling pathway is a highly conserved pathway that participates in various fundamental processes, including cell proliferation, differentiation, cell-fate determination, and stem cell maintenance [9]. Research has shown that the saponins of *Panax notoginseng* can improve wound healing in hyperglycemic rats via promoting endothelial cell proliferation, invasion, migration, angiogenesis, suppressing cell apoptosis and oxidative damage, and activating the GSK-3β/β-catenin pathway [10]. These results indicate the role of saponins of *Panax notoginseng* in promoting inflammatory skin healing and improving skin barrier function.

Due to the absence of the stratum corneum barrier, traditional two-dimensional cultured epidermal cells are limited in evaluating the effect of active ingredients on the formation of the stratum corneum barrier. Ex vivo skin is subject to clinical ethics and donor variability issues, with limitations in data stability and availability [11]. EpiKutis^®^ is a Reconstructed Human Epidermis (RHE) model that is a kind of highly biomimetic skin equivalent with natural skin gene expression, tissue structure, and metabolic activities formed by using induced pluripotent stem cells or keratinocytes and fibroblasts derived from adult stem cells as seed cells and differentiated by air–liquid surface culture technology [12]. In recent years, with the implementation of animal replacement tests in Europe and the United States, and the extensive research and application of RHE, the application of RHE has gradually expanded from laboratory research models to post-injury healing, immune metabolism, and inflammatory skin disease treatment, local toxicity following skin exposure, and drug testing [13]. A Sodium dodecyl sulfate (SDS)-stimulated EpiKutis^®^ epidermal model can induce the thickening and relaxation of the stratum corneum, showing the impairment of barrier function [14]. WY-14643, a peroxisome proliferator-activated receptor (PPAR) agonist with anti-inflammatory effects, can increase the expression of filaggrin and other structural proteins in the skin and has beneficial effects on skin homeostasis and the integrity of keratinocytes. This makes it a positive control drug for studying filaggrin-related skin diseases and skin barrier repair [15]. Jing et al. constructed a model capable of simulating surfactant-induced barrier damage in contact dermatitis by stimulating EpiKutis^®^ with surfactant SDS and evaluated the barrier repair effect of active ingredients [16]. As a highly biomimetic in vitro skin equivalent, RHE provides convenient and reliable research tools for studying the mechanism of skin barrier injury and the screening of barrier repair drugs.

Multi-omics analysis methods based on systems biology, including genomics, transcriptomics, proteomics, and metabolomics, play an indispensable role in decoding the interaction between TCMs and disease and the therapeutic mechanism of TCMs active ingredients [17]. Studies have shown that the combined analysis of the transcriptome and proteome can obtain gene and protein sets with the same or opposite expression trend. Functional enrichment analysis of different sets can further explore the key genes and proteins that regulate biological phenotypes. The combined analysis of metabolomics and proteomics is conducive to the functional interpretation of proteome data. By analyzing the enzymes or enzymatic pathways that regulate the changes in metabolites in proteomics, it is helpful to better understand the metabolomics data [18]. The integrated analysis of multi-omics can better explain the molecular mechanism of disease and the multi-target regulation mechanism of TCMs, and more intuitively understand the upstream and downstream regulatory relationship between metabolites, enzymes, and genes.

In this study, we for the first time integrated an RHE model with a systems biology approach—encompassing transcriptomics, proteomics, and lipid metabolomics—to investigate the efficacy and mechanism of a high-purity total saponins of *Panax notoginseng* (TSPN) in repairing skin barrier damage. Initially, the safety of TSPN for transdermal administration was evaluated in accordance with OECD guidelines to confirm that TSPN itself is non-irritating on EpiKutis^®^. Then we assessed the repair effect of TSPN on SDS-induced EpiKutis^®^ barrier injury through histological staining and immunofluorescence of barrier-related proteins. Subsequently, multi-omics analysis of these EpiKutis^®^ tissues was performed to identify genes, proteins, and lipid metabolites that trended to be expressed after TSPN intervention. Finally, the key proteins and lipid metabolites closely related to lipid metabolism for barrier repair were screened by multi-omics network analysis to clarify the mechanism of TSPN in repairing skin barrier damage.

## 2. Results

### 2.1. In Vitro Irritation Evaluation of TSPN

**Figure 1:**
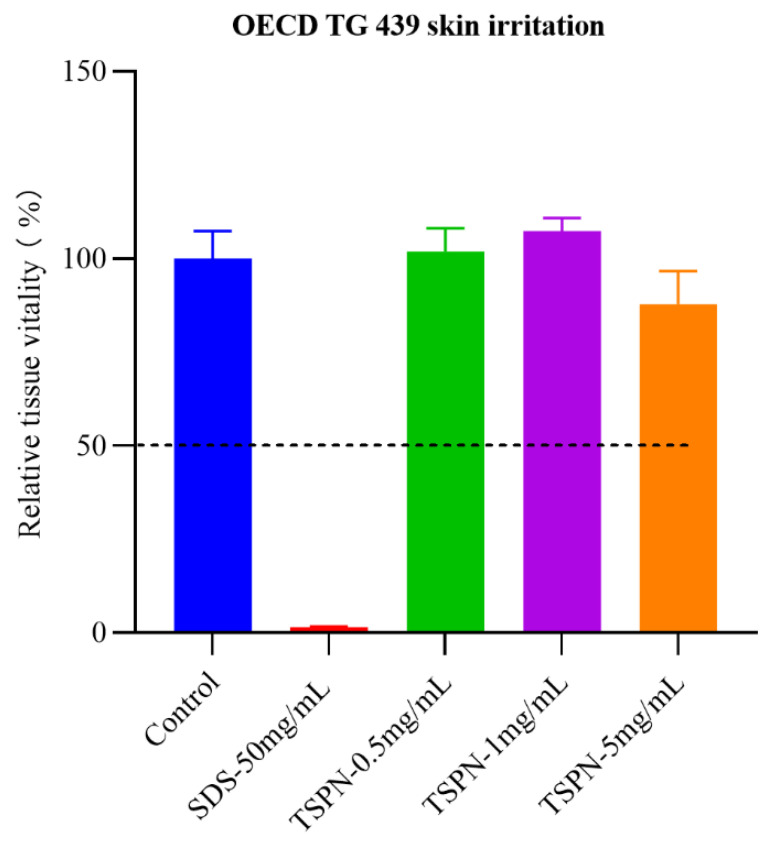
The EpiKutis^®^ skin tissue viability after TSPN stimulation.

The irritation results shown in Figure 1: SDS (50 mg/mL) was used as a positive control, tissue viability decreased to ≤20%, and was judged as an irritant. PBS was used as a negative control, and tissue viability was 100.00 ± 7.38%, which was judged as non-irritant. When the concentration of TSPN was 0.5, 1, and 5 mg/mL, the tissue vitality value was 87.7 ± 8.9~107.3 ± 3.4%, which was judged as non-irritant. The collective data classified TSPN as “Non-Irritant” (GHS Category 3) under UN GHS criteria. According to the results, 1 mg/mL TSPN was employed for the subsequent experiments.

### 2.2. Repair Efficacy of TSPN on SDS-Induced Epidermal Barrier Injury Model

**Figure 2:**
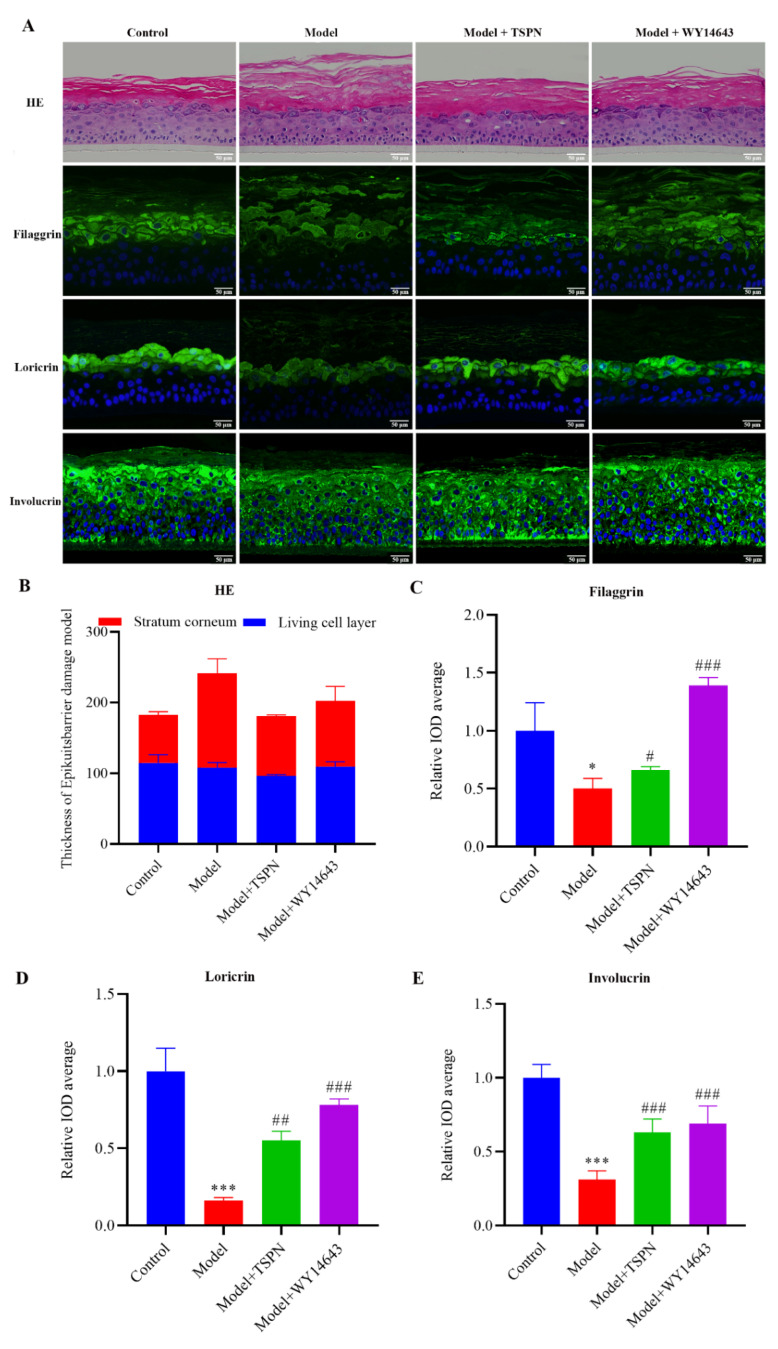
Repair efficacy of TSPN on SDS-induced epidermal barrier injury model. (**A**) skin stratum corneum morphology and the expression of barrier-related functional proteins after 1 mg/mL TSPN treatment. Nuclei are stained with DAPI (blue). The matrix components are stained with eosin (red). Functional proteins are marked in green. (**B**) Thickness of EpiKutis^®^ barrier damage model. (**C**) The relative IOD values of Filaggrin. (**D**) The relative IOD values of Loricrin. (**E**) The relative IOD values of Involucrin. Data presented as mean ± SD from at least three independent experiments. *p* values were determined by One-Way ANOVA (multiple comparisons), * *p* < 0.05, *** *p* < 0.001 vs. Control, ^#^ *p* < 0.05, ^##^ *p* < 0.01, ^###^ *p* < 0.001 vs. Model.

**Figure 3:**
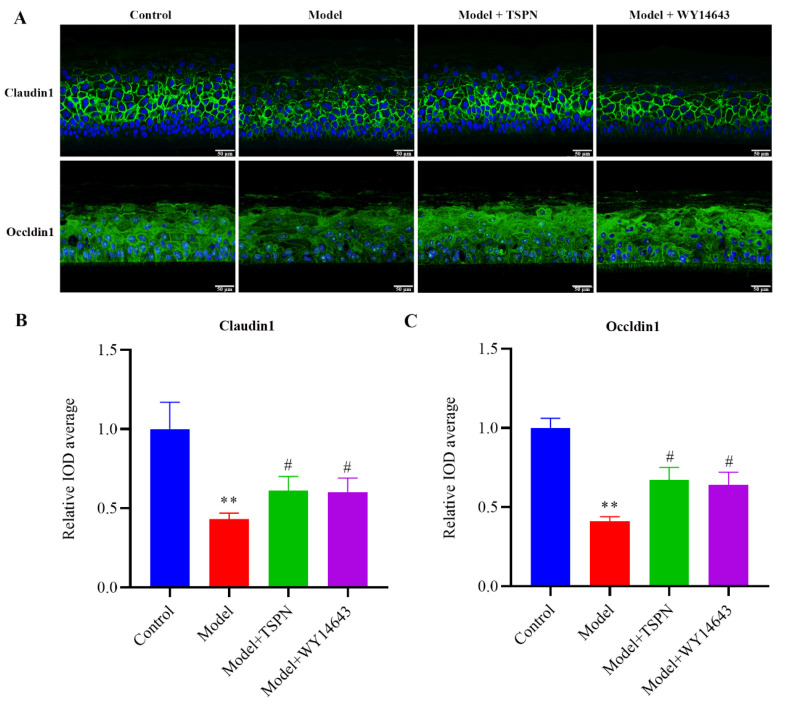
Repair efficacy of TSPN on SDS-induced epidermal barrier injury model. (**A**) The expression of tight junction proteins after 1 mg/mL TSPN treatment. Nuclei are stained with DAPI (blue). Tight junction proteins are marked in green. (**B**) The relative IOD values of Claudin1. (**C**) The relative IOD values of Occldin1. Data presented as mean ± SD from at least three independent experiments. *p* values were determined by One-Way ANOVA (multiple comparisons), ** *p* < 0.01 vs. Control, ^#^ *p* < 0.05 vs. Model.

To evaluate the reparative effects of TSPN on surfactant-induced epidermal barrier impairment, an SDS-induced EpiKutis^®^ skin model was established. As demonstrated in Figure 2A,B, the model group exhibited pronounced epidermal abnormalities compared to the control group, including hyperkeratosis with disorganized stratum corneum (SC) arrangement and reduced viable epidermal thickness, indicative of structural barrier impairment. Notably, 1 mg/mL TSPN treatment effectively attenuated SDS-induced SC thickening and restored the compactness of keratinocyte stratification, consistent with the therapeutic effects observed in the positive control group. In order to further investigate the effect of TSPN on skin barrier function, tissue immunofluorescence staining was used to detect barrier function-related proteins, and the results are shown in Figure 2 and Figure 3. Compared with the control group, the expression levels of Filaggrin (FLG), Involucrin (INV), and Loricrin (LOR), which are characteristic proteins of the SC, were decreased in the model group (*p* < 0.05). The expressions of tight junction proteins Claudin1 and Occldin1 were also significantly decreased (*p* < 0.05) in the model group. Treatment with TSPN significantly restored the expression of barrier-related proteins, indicating the reparative effects of TSPN on barrier-related functional proteins. In summary, the repair effect of TSPN on the SDS-induced epidermal barrier injury model was confirmed from the aspects of skin stratum corneum morphology and the expression of barrier-related functional proteins.

### 2.3. Transcriptomics Identified the Genes and Pathways Related to the Effect of TSPN on SDS-Induced Epidermal Barrier Injury Model

**Figure 4:**
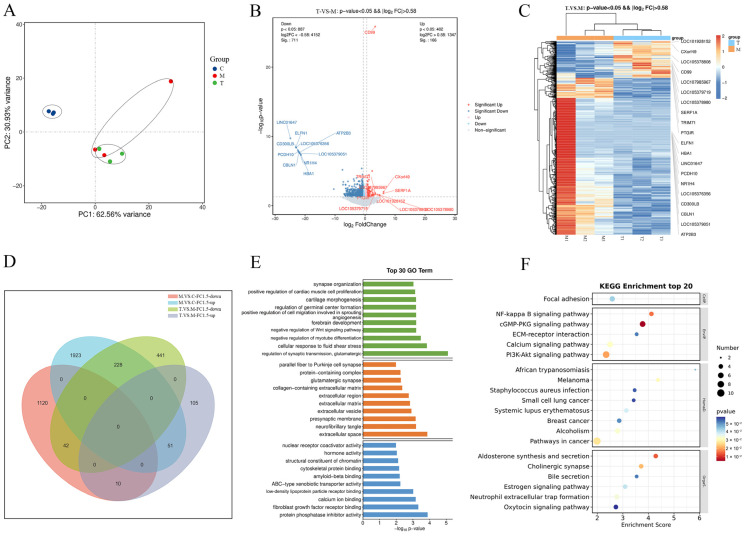
Transcriptomics identified the genes and pathways related to the effect of TSPN on the SDS-induced epidermal barrier injury model. (**A**) PCA score plots from the Control, Model, and TSPN groups. (**B**) The volcano plot of DEGs between the model group and the TSPN group. (**C**) Heat map analysis showed the difference between the model group and the TSPN group in the expression of genes through the color of the block. (**D**) Venn diagram shows the differentially expressed genes among the Model and TSPN groups, exhibiting opposite trends. (**E**) GO biological processes analysis for 238 genes with opposite expression trends in the three groups. (**F**) KEGG pathway analysis for 238 genes with opposite expression trends in the three groups.

To investigate the changes in mRNA expression in the EpiKutis^®^ skin following 1 mg/mL TSPN intervention, we conducted transcriptome sequencing of nine samples (the control, model, and TSPN groups) using the Illumina Novaseq^TM^ 6000 platform. The cDNA library sequencing conducted on the Illumina high-throughput sequencing platform yielded 62.51 G clean reads after rigorous quality control procedures. The average Q30 value was 95.86~96.27%, and the average GC content was 48.71%. To identify transcriptomic changes, we defined differentially expressed genes (DEGs) with |log2 fold change| ≥ 0.58 and a *p*-value of < 0.05. The PCA score plot of the transcriptome data showed that the different groups could be clearly separated (Figure 4A). The volcano plot analyses revealed significant differences among the control, model, and TSPN groups. Specifically, 3374 genes were differentially expressed between the control and model groups, with 2202 upregulated and 1172 downregulated (Figure S2 and Supplementary Material S1). Additionally, 877 genes differed between the TSPN and model groups, including 166 upregulated and 711 downregulated genes (Figure 4B and Supplementary Material S2). Z-score normalization was applied to all DEGs, and a clustering heat map was generated to illustrate the expression levels, with red squares indicating high expression and blue squares indicating low expression (Figure 4C and Figure S3). The Venn diagram analysis method was used to screen out the DEGs with opposite expression trends. As shown in Figure 4D, 228 DEGs were differentially expressed across all three groups, exhibiting opposite trends of first increasing and then decreasing. Additionally, 10 DEGs exhibited opposite trends of first decreasing and then increasing (Supplementary Material S3). To elucidate the functional roles of 238 DEGs with opposite expression trends, GO enrichment analysis identified 1312 pathways, with the top 30 displayed in Figure 4E. The analysis of biological processes indicated that DEGs with opposite expression trends were significantly enriched in pathways related to the regulation of synaptic transmission, glutamatergic, negative regulation of myotube differentiation, and regulation of synaptic transmission, glutamatergic. The analysis of cellular components indicated that DEGs with opposite expression trends were significantly enriched in pathways related to the extracellular matrix, collagen-containing extracellular matrix, and protein-containing complex. The analysis of molecular function indicated that DEGs with opposite expression trends were significantly enriched in pathways related to fibroblast growth factor receptor binding, cytoskeletal protein binding, and calcium ion binding. Moreover, KEGG pathway analysis yielded 65 pathways, with the top 20 illustrated in Figure 4F. Among these findings, the PI3K-Akt signaling pathway, Calcium signaling pathway, Focal adhesion, and ECM-receptor interaction were involved in the main biological processes of TSPN.

### 2.4. Proteomics Identified the Protein and Pathways Related to the Effect of TSPN on SDS-Induced Epidermal Barrier Injury Model

**Figure 5:**
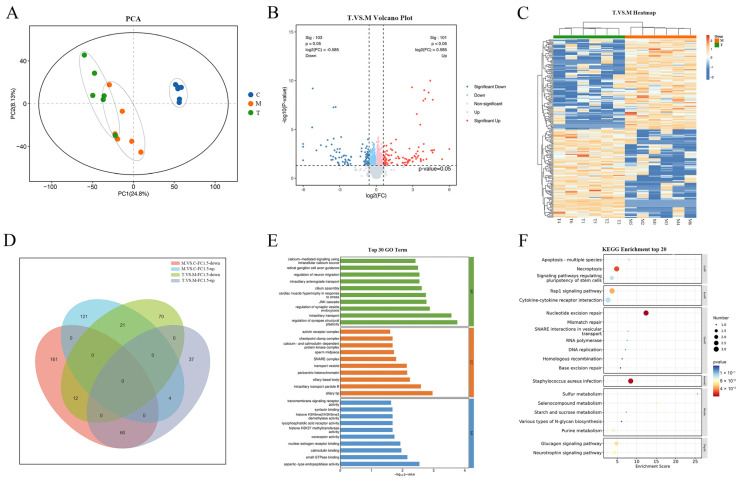
Proteomics identified the proteins and pathways related to the effect of TSPN on the SDS-induced epidermal barrier injury model. (**A**) PCA score plots from the Control, Model, and TSPN groups. (**B**) The volcano plot of DEPs between the Model group and the TSPN group. (**C**) Heat map analysis showed the difference between the Model group and the TSPN group in the expression of proteins through the color of the block. (**D**) Venn diagram shows the DEPs among the Model and TSPN groups, exhibiting opposite trends. (**E**) GO biological processes analysis for 81 DEPs with opposite expression trends in the three groups. (**F**) KEGG pathway analysis for 81 DEPs with opposite expression trends in the three groups.

To identify differential proteins in EpiKutis^®^ skins after TSPN treatment, we conducted proteomic sequencing of 18 samples (the control, model, and TSPN groups) using the iTRAQ-based quantitation proteomic strategy. To identify protein changes, we defined differentially expressed proteins (DEPs) with |log2 fold change| ≥ 0.58 and a *p*-value of <0.05. The PCA score plot of the proteomic data showed that the different groups could be clearly separated (Figure 5A). The volcano plot analyses revealed significant differences among the control, model, and TSPN groups. Specifically, 379 DEPs were differentially expressed between the control and model groups, with 146 upregulated and 233 downregulated (Figure S4 and Supplementary Material S4). Additionally, 204 DEPs differed between the TSPN and model groups, including 101 upregulated and 103 downregulated proteins (Figure 5B and Supplementary Material S5). Z-score normalization was applied to all DEPs, and a clustering heat map was generated to illustrate the expression levels, with red squares indicating high expression and blue squares indicating low expression (Figure 5C and Figure S5). The Venn diagram analysis method was used to screen out the DEPs with opposite expression trends. As shown in Figure 5D, 21 DEPs were differentially expressed across all three groups, exhibiting opposite trends of first increasing and then decreasing. Additionally, 60 DEPs exhibited opposite trends of first decreasing and then increasing (Supplementary Material S6). To elucidate the functional roles of 81 DEPs with opposite expression trends, GO enrichment analysis identified 773 pathways, with the top 30 being displayed in Figure 5E. The analysis of biological processes indicated that DEPs with opposite expression trends were significantly enriched in pathways related to the regulation of synapse structural plasticity, JNK cascade, and calcium-mediated signaling using an intracellular calcium source. The analysis of cellular components indicated that DEPs with opposite expression trends were significantly enriched in pathways related to transport vesicle, calcium- and calmodulin-dependent protein kinase complex, and activin receptor complex. The analysis of molecular function indicated that DEPs with opposite expression trends were significantly enriched in pathways related to lysophosphatidic acid receptor activity, calmodulin binding, and transmembrane signaling receptor activity. Moreover, KEGG pathway analysis yielded 65 pathways, with the top 20 illustrated in Figure 5F. Among these findings, the necroptosis, cytokine–cytokine receptor interaction, neurotrophin signaling pathway, apoptosis-multiple species pathway, various types of N-glycan biosynthesis, and signaling pathways regulating pluripotency of stem cells were involved in the main biological processes of TSPN.

### 2.5. Metabolomics Identified the Lipid Metabolites and Pathways Related to the Effect of TSPN on SDS-Induced Epidermal Barrier Injury Model

**Figure 6:**
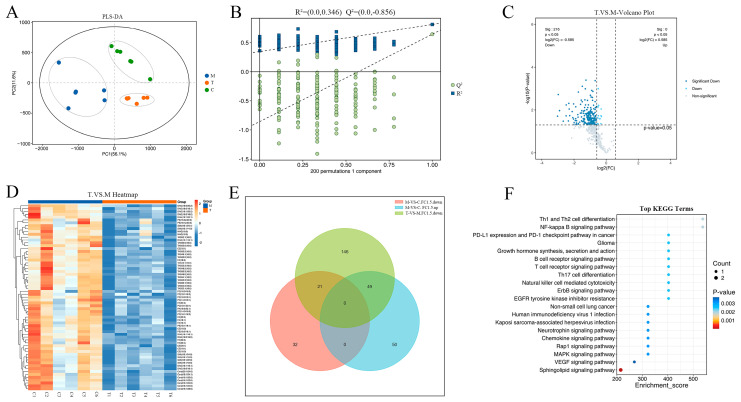
Metabolomics identified the lipid metabolites and pathways related to the effect of TSPN on **the** SDS-induced epidermal barrier injury model. (**A**) PLS-DA score plots from the Control, Model, and TSPN groups. (**B**) PLS-DA model validation diagram. (**C**) The volcano plot of DEPs between the Model group and the TSPN group. (**D**) Heat map analysis showed the difference between the Model group and the TSPN group in the expression of lipid metabolites through the color of the block. (**E**) Venn diagram shows the differentially expressed lipid metabolites among the Model and TSPN groups, exhibiting opposite trends. (**F**) KEGG pathway analysis for 49 lipid metabolites with opposite expression trends in the three groups.

To identify differential lipid metabolites in EpiKutis^®^ skins after TSPN treatment, we conducted lipid metabolomics of 18 samples (the control, model, and TSPN groups) using the ESI-Q TRAP-MS/MS technology combined with statistical methods. The Compound Discoverer 3.0 software was used for MS data analysis, and 474 metabolites were found by the Lipid MAPS, MZ Cloud, Lipid Blast, and ChemSpider databases. Then the MS data were imported into SIMCA-P V14.1 for multivariate statistical analyses, such as PCA, PLS-DA, and OPLS-DA. PCA models are used to explore differences in the overall data between different groups, and are also used to investigate instrument stability for large-scale sample analysis by the degree of QC sample repeatability. The PCA score plot from LC-MS demonstrated that the different groups could be clearly separated, and all of the QC samples were closely correlated (Figure S6), indicating excellent repeatability of samples and instruments, which ensures data reliability. The reliability and the degree of overfitting for the multivariate statistical analysis model were checked using the Permutation plot of the PLS-DA model (Figure 6A,B). The values of R^2^ X, R^2^ Y, and Q^2^ of the PLS-DA model in LC-MS were 0.763, 0.603, and 0.336, respectively, indicating that the model had excellent predictive power. As seen in Figure 6A, all of the different groups could be clearly separated, and the skin lipid metabolic profiles of the TSPN group were closer to the control group and further from the model group, suggesting that the skin lipid metabolic disturbances induced by SDS were reversed after TSPN treatment. To identify lipid metabolite changes, we defined differentially expressed metabolites (DEMs) with |log2 fold change| ≥ 0.58 and a *p*-value of <0.05. The volcano plot shows that 152 lipid metabolites were differentially expressed between the control and model groups, with 99 upregulated and 53 downregulated (Figure S7 and Supplementary Material S7). Additionally, 216 lipid metabolites differed between the TSPN and model groups, including 0 upregulated and 216 downregulated lipid metabolites (Figure 6C and Supplementary Material S8). Z-score normalization was applied to all DEMs, and a clustering heat map was generated to illustrate the expression levels, with red squares indicating high expression and blue squares indicating low expression (Figure 6D and Figure S8). The Venn diagram analysis method was used to screen out the DEMs with opposite expression trends. As shown in Figure 6E, 49 lipid metabolites were differentially expressed across all three groups, exhibiting opposite trends of first increasing and then decreasing (Supplementary Material S9). To elucidate the relevant signaling pathways of 49 DEMs with opposite expression trends, KEGG pathway analysis yielded 65 pathways, with the top 20 illustrated in Figure 6F. Among these findings, the Sphingolipid signaling pathway, VEGF signaling pathway, MAPK signaling pathway, Rap1 signaling pathway, Chemokine signaling pathway, Neurotrophin signaling pathway, and NF-kappa B signaling pathway were involved in the main biological processes of TSPN.

### 2.6. Multi-Omics Analysis Revealed the Key Targets of TSPN on SDS-Induced Epidermal Barrier Injury Model

**Figure 7:**
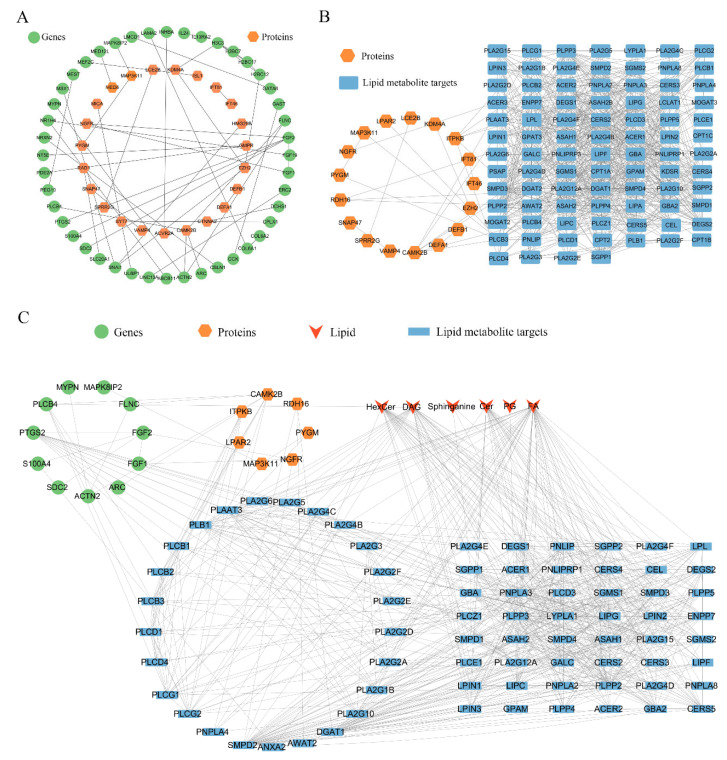
Multi-omics analysis revealed the key targets of TSPN treatment for skin barrier repair. (**A**) Transcription–Protein Interaction Network. (**B**) Protein–Lipid Metabolite Interaction Network. (**C**) Transcription–Protein–Lipid Metabolite Targets–Lipid Metabolite Interaction Network.

To elucidate the key targets for TSPN on the SDS-induced epidermal barrier injury model at the protein level, the STRING database (confidence score ≥ 0.7) was employed to retrieve experimentally validated interactions between 238 DEGs with opposite expression trends and 81 DEPs with opposite expression trends, followed by Cytoscape 3.10.2 software for network visualization to construct a Transcription–Protein Interaction Network. As shown in Figure 7A, among the 81 DEPs with opposite expression trends, only 23 DEPs had an interaction with the 44 DEGs of the transcriptome, and the top 10 most interconnected proteins with DEGs were NGFR, CAMK2B, PYGM, SPRR2G, GMPR, MAP3K11, EZH2, HMG20B, ACVR2A, and RAD1.

To further elucidate the potential connections between 81 DEPs with trend expression and 49 DEMs with trend expression, the Lipid Maps database was first employed to search for the enzymes and proteins that guide the production of differential lipid metabolites. Then it was used as the target of lipid metabolites and uploaded to the STRING database (confidence score ≥ 0.7) along with 81 DEPs with opposite expression trends to construct a Protein–Lipid metabolite Interaction Network. The 141 targets for information on lipid metabolites are shown in Supplementary Material S10. As shown in Figure 7B, 17 out of 81 DEPs with trend expression interact with the 88 targets of lipid metabolites, and the most interconnected proteins with DEPs were CAMK2B, ITPKB, RDH16, PYGM, NGFR, LPAR2, and MAPK3K11, which were the most interconnected proteins with the target of lipid metabolites. Further, the Transcription–Lipid metabolite Interaction Network was constructed through integrating 238 DEGs with opposite expression with 141 targets of lipid metabolites. As shown in Figure S9, 39 out of 238 DEGs with trend expression interact with the 88 targets of lipid metabolites, and the top10 most interconnected genes with the target of lipid metabolites were *FGF2*, *DGK1*, *UGT8*, *PTGS2*, *LIPC*, *ACTN2*, *GNG8*, *NR1H4*, *COL6A1*, and *MYPN*, which are the most interconnected genes with the target of lipid metabolites.

**Figure 8:**
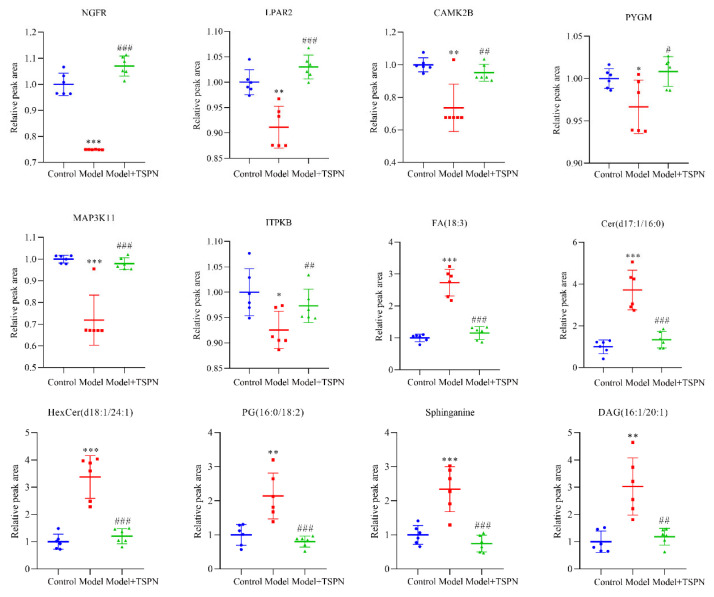
Comparison of relative peak areas of the key target proteins and lipid metabolites in multi-omics analysis with TSPN treatment. All data were expressed as mean ± SD (n = 8). * *p* < 0.05, ** *p* < 0.01, *** *p* < 0.001 compared with Control group. ^#^ *p* < 0.05, ^##^ *p* < 0.01, ^###^ *p* < 0.001 compared with Model group.

To elucidate the potential connections among DEGs with trend expression, DEPs with trend expression, the target of lipid metabolites, and DEMs with trend expression, we integrated the transcription–protein interaction network, protein–lipid metabolite interaction network, and transcription–lipid metabolite interaction network to construct a Transcription–Protein–Lipid metabolite targets–Lipid metabolite Interaction Network (Figure 7C). All the interaction nodes included 10 DEGs with trend expression, 7 DEPs with trend expression, 73 targets of lipid metabolites, and 6 lipid metabolites. The overlapping key target proteins and lipid metabolites were screened, including CAMK2B, ITPKB, RDH16, PYGM, NGFR, LPAR2, MAPK3K11, FA (18:3), Cer (d17:1/16:0), HexCer (d18:1/24:1), PG (16:0/18:2), Sphinganine, and DAG (16:1/20:1). As shown in Figure 8, these key target proteins and lipid metabolites showed significant reversal after TSPN intervention compared with the model group, which likely play pivotal roles in the skin barrier repair efficacy of TSPN.

### 2.7. Multi-Omics Analysis Revealed the Mechanism of Action of TSPN in the Treatment of SDS-Induced Epidermal Barrier Injury Model

**Figure 9:**
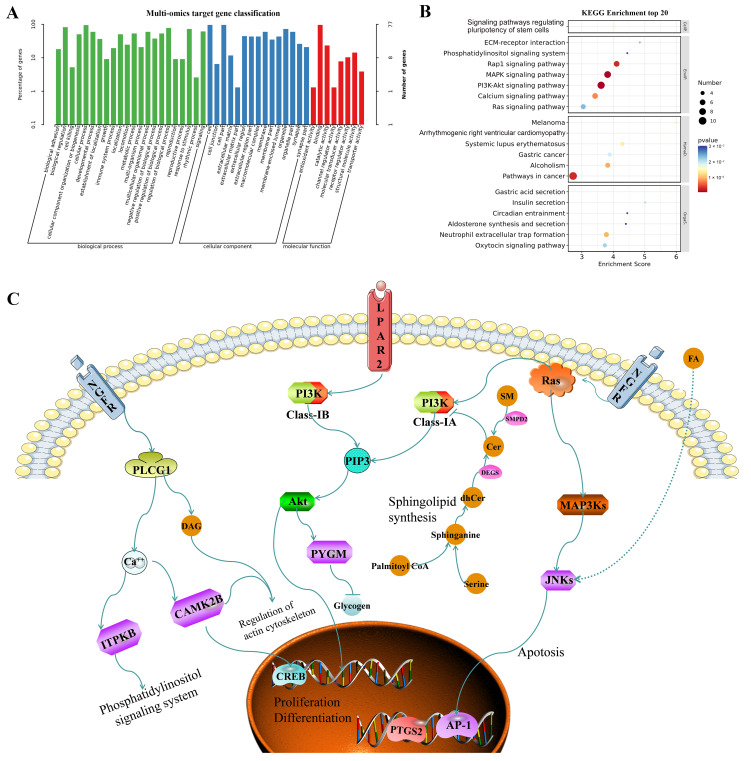
Multi-omics analysis revealed the mechanism of action of TSPN treatment for skin barrier repair. (**A**) GO classification analysis for the 77 key genes and proteins screened from multi-omics interaction network analysis. (**B**) KEGG pathway analysis for the 77 key genes and proteins screened from multi-omics interaction network analysis. (**C**) Schematic representation of TSPN regulating lipid metabolism disorders in a model of SDS-induced epidermal barrier injury.

In order to further explain the molecular mechanism of TSPN in repairing SDS-induced skin barrier damage, the 77 key genes and proteins screened from the Transcription–Protein Interaction Network, Transcription–Metabolic Interaction Network, and the Protein–Metabolic Interaction Network were selected as potential targets for multi-omics joint analysis (Supplementary Data S11), and then imported it into the DAVID database for GO and KEGG analysis. As shown in Figure 9A, these potential targets were grouped into 45 categories. The analysis of biological processes indicated that potential targets were significantly enriched in pathways related to biological adhesion, growth, immune system processes, and response to stimuli. The analysis of cellular components indicated that potential targets were significantly enriched in pathways related to cell junctions, extracellular matrix, membrane parts, and synapse parts. The analysis of molecular function indicated that potential targets were significantly enriched in pathways related to antioxidant activity, channel regulator activity, and receptor regulator activity. Moreover, KEGG pathway analysis yielded the top 20 illustrated in Figure 9B. Among these findings, the PI3K-Akt signaling pathway, the MAPK signaling pathway, the Calcium signaling pathway (regulating the pluripotency of stem cells), the ECM-receptor interaction, and the Phosphatidylinositol signaling system were involved in the main biological processes of TSPN. Through further integrating seven key target proteins and six lipid metabolites screened from the Transcription–Protein–Lipid metabolite targets–Lipid metabolite Interaction Network into the above 20 pathways, the final pathway map (Figure 9C) was constructed to display the correlation among the key target proteins and lipid metabolites visually and explain the multiple target mechanism of TSPN in the treatment of SDS-induced epidermal barrier injury model. According to the pathway map, multiple targets involved in multiple pathways were directly or indirectly related to the PI3K-AKT-mediated proliferation and differentiation pathway, MAPK-mediated apoptotic pathways, Sphingolipid synthesis, CAMK2B-mediated actin cytoskeleton regulation, and ITPKB-mediated phosphatidylinositol signaling system.

## 3. Discussion

*Panax notoginseng* saponins (PNS), as the main active ingredient of Panax, have been widely used in the percutaneous treatment of wound healing and skin diseases. However, the efficacy and mechanism of barrier repair remain unclear. The establishment of a skin barrier damage model is a key step in skin science research, which is helpful in evaluating the barrier repair effect of the active ingredients of TCMs and in deeply understanding the mechanism of skin barrier repair [19]. The two-dimensional cultured HaCaT cells showed the absence of cuticle structure and function, while the three-dimensional cultured recombinant human epidermal model could reproduce the physiological structure and barrier-related characteristic proteins of the natural skin epidermal layer in vitro [13]. The barrier function of the skin largely depends on the correct adhesion between keratinocytes. FLG and LOR are characteristic proteins that constitute the stratum corneum barrier, and the tight junction proteins Claudin1 and Occldin1 play a key role in intercellular tight junctions [20]. The anionic surfactant SDS is widely applied and used as a barrier damage model irritant in in vitro studies [21]. In this study, we used the SDS-induced 3D epidermal barrier damage model to investigate the repair efficacy of the TSPN. Our findings demonstrate that treatment with the TSPN significantly attenuated SDS-induced stratum corneum thickening and restored the compactness of keratinocyte stratification, increased the expression of stratum corneum barrier proteins and intercellular tight junction proteins. In summary, the repair effect of the TSPN on the skin barrier is mainly reflected in the enhancement of the barrier structure of the stratum corneum and epidermal intercellular adhesion.

Given the unique multi-component, multi-target, and multi-pathway therapeutic characteristics of TCMs, this study used a multi-omics joint analysis method to explain the multi-target pathways and associations of the TSPN at the level of gene, protein, and lipid metabolites, and identify the key targets and key pathways that exert their barrier repair effect. In this study, for the first time, we performed a multi-omics analysis of a 3D RHE model from the blank group, the model group, and the TSPN treatment group using transcriptome, proteome, and lipid metabolomics technology. A total of 238 differentially expressed genes, 81 differentially expressed proteins, and 49 lipid metabolites were identified. It is worth noting that due to the complexity of biological systems, there are often inconsistencies among mRNA expression, protein abundance, and metabolite kinetics [22]. Protein–Protein Interaction Networks (PPI) are often used to analyze the interaction of proteins in biological systems. A PPI network can integrate multi-omics data, establish a gene–protein–metabolite cascade network, and mine core regulatory molecules [23]. In this study, seven key target proteins and six lipid metabolites were screened out by constructing the of transcription–protein–lipid metabolite target–lipid metabolite interaction network, including CAMK2B, ITPKB, RDH16, PYGM, NGFR, LPAR2, MAPK3K11, FA (18:3), Cer (d17:1/16:0), HexCer (d18:1/24:1), PG (16:0/18:2), Sphinganine, and DAG (16:1/20:1). According to our results, these protein and lipid metabolites were involved in the PI3K-AKT-mediated proliferation and differentiation pathway, MAPK-mediated apoptotic pathways, Sphingolipid synthesis, CAMK2B-mediated actin cytoskeleton regulation, and ITPKB-mediated phosphatidylinositol signaling system, which highlight the complex and multifaceted role of TSPN in repairing the skin barrier by regulating lipid metabolism.

The intracellular calcium gradients and levels of the calcium-binding protein CAMK2B are reduced in keratinocytes during skin barrier damage [24]. CAMK2B expression was reduced in the imiquimod-induced psoriasis mouse model, which confirmed that the SDS-induced thickening of the stratum corneum in the 3D skin model was correlated with CAMK2B expression [25]. Lysophosphatidic acid (LPA) is a bioactive phospholipid that modulates numerous cellular responses, such as proliferation, survival, and migration, in the imiquimod-induced psoriasis mouse model [26]. LPA/LPAR1 signaling induces PGAM1 expression via the PI3K-AKT pathway and increases aerobic glycolysis, contributing to keratinocyte proliferation [27]. Glycogen phosphorylase (PYGM) is a key enzyme that regulates glucose homeostasis and energy metabolism plasticity, and is also a key molecule in skin regeneration [28]. Phosphatidylinositol metabolism plays a key role in keratinocyte terminal differentiation and stratum corneum barrier generation. The loss of epidermal phosphatidylinositol metabolizing enzymes leads to the dysregulation of intracellular calcium and the excessive activation of the MAPK pathway, thereby impairing the epidermal barrier function [29]. Inositol-Trisphosphate 3-kinase B (ITPKB) enzyme is involved in wound healing by regulating intracellular calcium influx; the deletion of the ITPKB gene in the skin leads to the interruption of calcium signal transduction and affects skin re-epithelialization [30]. The production of endogenous retinoid in epidermal keratinocytes is regulated by the retinol metabolism pathway, which can be used to improve epidermal hyperkeratosis. In the process of calcium-induced keratinocyte differentiation, retinol dehydrogenase 16 (RDH16) is increased in keratinocytes, while excessive calcium mobilization leads to excessive proliferation and permeability dysfunction of the epidermis, and the expression of RDH16 and retinoid is decreased [31,32]. NGFR is a 75 kDa highly conserved transmembrane glycoprotein that promotes fibroblast and keratinocyte proliferation, extracellular matrix component expression and secretion, angiogenesis, and myofibroblast differentiation [33]. NGFR promotes wound re-epithelialization by activating PI3K/Akt-Rac1-JNK and ERK signaling pathways [34]. Free fatty acids can promote the production of lipid droplets in sebaceous cells, mediate the production of caspase3, and induce apoptosis [35]. The production of ceramide in the stratum corneum is related to the process of keratinization [36]. Due to the abnormal epidermal differentiation induced by SDS, the thickness of the stratum corneum is increased, and the ceramide content increases. The abnormally increased ceramide acts as a second messenger, which can activate caspase3 and induce cell apoptosis through the PI3K-AKT pathway [37]. Phosphatidylglycerol (PG) plays a key role in modulating keratinocyte proliferation, which may prove useful for treating skin diseases characterized by excessive or insufficient proliferation [38]. PG species comprising polyunsaturated fatty acids are most effective at inhibiting proliferation, whereas PG species containing monounsaturated and saturated fatty acids are best at promoting keratinocyte growth [39,40]. Altered levels of sphingosine, sphinganine, and their ceramides in atopic dermatitis are related to dysfunctional skin barrier function. As compared to healthy skin, the levels of sphingosine and sphinganine are elevated in atopic dermatitis [41]. Keratinocyte differentiation is partly regulated by protein kinase C (PKC), which has two common regions, diacylglycerol (DAG)-binding C1 domain and Ca^2+^-binding C2 domain, in the regulatory domain. Calcium, phosphatidylserine, and DAG are required for their activation [42]. TCMs have the unique advantages of multi-component, multi-target, and multi-pathway overall regulation. The discovery of its pharmacological material basis is currently a hot topic and core issue in the research of TCMs. Although this study has clearly identified the composition and content of the main active components of TSPN, the specific components responsible for its therapeutic effect and their respective contributions need to be further verified through experiments. It is noteworthy that although the RHE model is structurally similar to human skin, it still lacks certain functionalities, such as immune and neurological functions. The correlation between their overall molecular-level changes in multi-omics analyses and those in human skin requires further investigation. In this study, although these data confirm the TSPN’s combinatorial effects associated with barrier impairment, additional research is needed to fully align modern experimental results with long-standing ethnopharmacological practices.

## 4. Materials and Methods

### 4.1. Materials

The total saponins of *Panax notoginseng* (TSPN) were purchased from the Zelang Biotechnology Co., Ltd. (Nanjing, China). The quality control of the TSPN was monitored by high-performance liquid chromatography (HPLC) (Figure S1A,B). The constituents of TSPN include notoginsenoside R1 (content: 8.35%), ginsenoside Rg1 (content: 37.7%), ginsenoside Re (content: 3.66%), ginsenoside Rb1 (content: 36.1%), and ginsenoside Rd (content: 8.51%). In vitro EpiKutis^®^ Skin and EpiGrowth culture medium were procured from Boxi Biotech Co, Ltd. (Xi’an, China). MTT, WY14643, and Sodium dodecyl sulfate were obtained from Sigma Company (St. Louis, MO, USA). The 4% paraformaldehyde, hematoxylin, and eosin were obtained from the Beyotime Biotechnology company (Shanghai, China). PBS and paraformaldehyde were obtained from Solarbio company (Beijing, China). All organic reagents were obtained from Sinopharm Chemical Reagent Co, Ltd. (Taiyuan, China). Rabbit anti-filaggrin (FLG), rabbit anti-loricrin (LOR), rabbit anti-Involucrin (INV), rabbit anti-Occldin1, and rabbit anti-Claudin1 were purchased from Abcam company (Chicago, IL, USA). The 4′,6-diamidino-2-phenylindole (DAPI), goat anti-rabbit IgG-Alexa Fluor 488, and phalloidin-FITC were obtained from Thermo Fisher Scientific (Waltham, MA, USA).

### 4.2. In Vitro Skin Irritation

The in vitro skin irritation test was conducted to determine the TSPN’s application dosage. Skin irritation tests of TSPN were performed using EpiKutis^®^, a reconstructed human epidermal tissue model validated by OECD TG 439 in vitro skin irritation guidelines [43,44]. The procedure begins with pre-incubation of EpiKutis^®^ tissues at 37 °C and 5% CO_2_ for 24 h to stabilize viability. The 200 µL of the TSPN at 0.5, 1, and 5 mg/mL concentrations were applied topically to the epidermal surface and incubated for 30 min under controlled conditions. PBS was used as a negative control, and a 50 mg/mL SDS acted as a positive control (PC). Post-exposure, tissues were rinsed to remove residues and transferred to fresh medium for 24 h incubation. Cell viability was quantified via the MTT assay: tissues are incubated with 1 mg/mL MTT for 3 h, allowing for mitochondrial dehydrogenases to reduce MTT to formazan. The formazan crystals are extracted using isopropanol, and absorbance is measured spectrophotometrically at 570 nm (reference: 650 nm). A test substance is classified as an irritant if the mean relative viability of three replicates is ≤50% of negative controls. Non-irritants exhibit viability > 50%.

### 4.3. SDS-Induced EpiKutis^®^ Barrier Injury Model Construction and Drug Intervention

The reconstructed human epidermal model (EpiKutis^®^) was cultured under air–liquid interface conditions at 37 °C and 5% CO_2_ for 24 h to stabilize barrier function. SDS was dissolved in phosphate-buffered saline (PBS) to prepare a 1 mg/mL solution, a concentration validated for inducing reproducible barrier disruption in prior studies. The EpiKutis^®^ tissues were randomly divided into 4 groups, with 3 in each group: (1) Control group (untreated tissues), (2) Model group (SDS-damaged tissues without intervention), (3) TSPN-treated group (Model + TSPN), and (4) WY14643-treated group (Model + WY14643). The 25 µL of the SDS at 1 mg/mL concentration was topically applied to the epidermal surface of EpiKutis^®^ tissues for 0.5 h to obtain the SDS-induced EpiKutis^®^ barrier injury model. And then, the prepared 25 μL of 1 mg/mL TSPN and 50 μM WY14643 were topically applied to the epidermal surface of EpiKutis^®^ tissues for 24 h, followed by gentle rinsing with PBS to remove residual irritants.

### 4.4. Histological Analysis

For Hematoxylin-Eosin (H&E) Staining, EpiKutis^®^ tissues were fixed in 4% paraformaldehyde for 24 h at 4 °C, dehydrated through graded ethanol series, and embedded in paraffin. Serial sections (5 µm thickness) were cut using a rotary microtome and mounted onto glass slides. Deparaffinized sections were stained with hematoxylin for 8 min, rinsed in tap water, and differentiated in 1% acid ethanol. Counterstaining was performed with eosin Y for 2 min. Sections were dehydrated, cleared in xylene, and mounted with neutral balsam. Morphological alterations were analyzed under a light microscope using Image-J (v 1.8.0.345). The thickness of the epidermis was measured using the measurement tool of the Image-J (v 1.8.0.345). Three regions were randomly selected from each sample to measure the living cell layer with nuclei and the dead cell layer without nuclei, and the average thickness was used for statistical analysis.

For immunofluorescence staining, cryosections were permeabilized with 0.1% Triton X-100 for 15 min and blocked with 5% bovine serum albumin (BSA) for 1 h. Primary antibodies against barrier-related proteins (Filaggrin [1:200], Claudin1 [1:100], Loricrin [1:300], Occldin1 [1:100], Involucrin [1:200]) were applied overnight at 4 °C. After PBS washes, sections were incubated with Alexa Fluor 488/594-conjugated secondary antibodies (1:500, Invitrogen, Carlsbad, CA, USA) for 1 h at room temperature. Nuclei were counterstained with 1 µg/mL DAPI. Fluorescence signals were captured using a confocal microscope (Zeiss LSM 900), and mean fluorescence intensity (MFI) was quantified using ZEN software (v 2.1). The integrated optical density (IOD) values are obtained using Image-J software. Relative IOD values are commonly employed to compare the differences in immunofluorescence staining intensities among different samples. The calculation method is to divide the IOD of a specific area by the total IOD of that area, and then divide the result by the area of that region.

### 4.5. Transcriptomic Sequencing

Transcriptomic sequencing and analysis were conducted by OE Biotech Co., Ltd. (Shanghai, China). EpiKutis^®^ tissues (n = 3 per group) were harvested 24 h post-SDS exposure or TSPN intervention and immediately snap-frozen in liquid nitrogen. Total RNA was extracted using TRIzol^®^ Reagent (Vazyme, Nanjing, China), followed by DNase I treatment to eliminate genomic DNA. RNA integrity was verified via Agilent Bioanalyzer 2100 (Waldbronn, Germany). Stranded mRNA libraries were prepared using the VAHTS Universal V6 RNA-seq Library Prep Kit (Vazyme, Nanjing, China). Raw reads in fastq format were processed using FASTQC software (v 0.12.1) to remove low-quality reads, obtaining clean reads for subsequent analysis. The clean reads were mapped to the reference genome using HISAT2. The FPKM of each gene was calculated, and the read counts of each gene were obtained by HTSeq-count. PCA was performed using R (v 3.2.0) to evaluate the biological duplication of samples. Differentially expressed genes (DEGs) were identified using DESeq2 (v1.38.3) with thresholds of fold change > 1.5 or fold change < 0.66 and the adjusted *p*-value < 0.05. Volcano diagram and Venn diagram were performed using R (v 3.2.0) to show the DEGs with trend callback. Hierarchical clustering analysis of DEGs was performed using R (v 3.2.0) to show the expression patterns of genes in different groups and samples. Based on the hypergeometric distribution, GO and KEGG pathway enrichment analysis of DEGs were performed to screen the significant enriched terms using R (v 3.2.0), respectively.

### 4.6. Proteomic Sequencing

Proteomic sequencing and analysis were conducted by OE Biotech Co., Ltd. (Shanghai, China). EpiKutis^®^ tissues (n = 6 per group) were collected 24 h post-intervention, flash-frozen in liquid nitrogen, and homogenized in RIPA buffer supplemented with protease inhibitors. Total protein concentrations were determined via BCA assay, and 50 µg of protein per sample was subjected to reduction (10 mM dithiothreitol, 30 min, 56 °C), alkylation (20 mM iodoacetamide, 20 min, dark), and tryptic digestion (1:50 enzyme-to-protein ratio, 37 °C, 16 h). Digested peptides were desalted using C18 StageTips and labeled with TMTpro 16plex reagents following manufacturer guidelines to enable multiplexed quantification. Labeled peptides were fractionated via high-pH reversed-phase HPLC into 12 fractions to reduce complexity. LC-MS/MS analysis was performed on a hybrid TIMS quadrupole TOF mass spectrometer (Bruker, Bremen, Germany) coupled to an EASY-nLC 1200 system (Thermo, Waltham, MA, USA), utilizing a 120 min acetonitrile gradient (2–35%) over a C18 column (75 µm × 25 cm, 2 µm particles). MS data were acquired in data-dependent acquisition mode with a full MS scan range of 350–1500 *m*/*z*. Spectronaut^®^ was used to search all of the raw data thoroughly against the sample protein database. The database search was performed with Trypsin digestion specificity. Alkylation on cysteine was considered a fixed modification in the database searching. For DIA data, the quantification FDR was also set to 0.05. Differentially expressed proteins (DEPs) were identified via limma (R v4.4.0) with thresholds: fold change > 1.5 or fold change < 0.66, *p*-value < 0.05. Volcano plots highlighted significant DEPs, while heatmaps visualized Z-score normalized intensities clustered by Euclidean distance. The Venn diagram analysis method was used to screen out the DEPs with opposite expression trends. GO and KEGG pathway analyses were performed using clusterProfiler (v 4.9.0).

### 4.7. Lipidomic Analysis

Lipidomic analysis was conducted by OE Biotech Co., Ltd. (Shanghai, China) using validated protocols. EpiKutis^®^ tissues (n = 6 per group) were harvested 24 h post-SDS exposure or TSPN intervention, snap-frozen in liquid nitrogen, and homogenized in pre-cooled methanol:methyl tert-butyl ether (1:3 *v*/*v*) containing isotope-labeled internal mix standards obtained from Avanti Polar Lipids and Sigma-Aldrich (St. Louis, MO, USA). After vortexing for 30 s and sonication for 10 min, samples were centrifuged at 13,000× *g* rpm and 4 °C for 10 min, and the organic phase was collected and lyophilized in a centrifugal vacuum evaporator, and lipids were reconstituted in 200 µL isopropanol:acetonitrile (1:1 *v*/*v*) for subsequent analyses. The quality-control sample was prepared by mixing equal volumes of aliquots of the supernatants from all samples. Lipid separation was performed on a UHPLC system using a HSS T3 (2.1 × 100 mm, 1.7 µm; Waters) with a 25 min gradient at 0.3 mL/min. The mass spectrometry (MS) system was QTRAP 6500+ (SCIEX, Waltham, MA, USA) equipped with an IonDrive Turbo V source, and the assay was performed in the negative/positive-ion working mode with a time-scheduled multiple reaction monitoring method. The source condition was as follows: curtain gas: 35 psi; medium: CAD; IS: 4.5 kV/þ5.5 kV; Gas1: 40 psi; Gas2: 45 psi. QC samples, pooled from all groups, were injected every 6 runs to monitor system stability. Raw data were processed using MS-DIAL (v4.9) for peak alignment, lipid identification (LIPID MAPS database), and quantification. Features with >30% RSD in QCs were excluded. PCA demonstrated an overview of sample distributions and analysis stability. OPLS-DA and PLS-DA facilitated the identification of differing metabolites between groups. To mitigate overfitting, cross-validation and 200 Response Permutation Tests assessed model quality. VIP values derived from the OPLS-DA model ranked each variable’s overall contribution to group discrimination. Subsequently, a two-tailed Student’s *t*-test confirmed the significance of inter-group differences in metabolites (DEMs). The altered metabolites were selected based on VIP values (VIP > 1), S-plot (cut-off value of *p* (corr) > 0.58 or *p* (corr) < −0.58), and *t*-test (*p* < 0.05).

### 4.8. Statistical Analysis

Statistical analysis was performed using GraphPad Prism 8.0, and data were expressed as mean ± standard deviation. Epidermal thickness and MFI values were expressed as mean ± SD. Statistical significance (*p* < 0.05) was determined via One-Way ANOVA followed by Tukey’s post hoc test.

## 5. Conclusions

In conclusion, this study confirmed that the 1 mg/mL TSPN can repair SDS-induced thickening and loosening of the stratum corneum by increasing the expression of barrier-associated proteins. Multi-omics studies suggest that the key targets of TSPN in barrier repair by regulating lipid metabolism include CAMK2B, ITPKB, RDH16, PYGM, NGFR, LPAR2, and MAPK3K11. TSPN exerts a barrier repair efficacy, potentially through the regulation of key signaling pathways, including PI3K-AKT-mediated proliferation and differentiation pathways, MAPK-mediated apoptotic pathways, Sphingolipid synthesis, CAMK2B-mediated actin cytoskeleton regulation, and the ITPKB-mediated phosphatidylinositol signaling system. This integrative strategy not only deepens the understanding of TSPN’s barrier repair mechanisms but also provides a novel framework for active ingredients of TCMs in the context of stratum corneum barrier dysfunction, highlighting the potential of RHE and multi-omics approaches in the study of active ingredients of TCMs.

## Data Availability

The transcriptomic data is available in the SRA database (accession number PRJNA1345721). The proteomics data is available in the iProX database (accession number PXD069658). The metabolites data analyzed is available from the corresponding author on reasonable request. The raw data supporting the conclusions of this article will be made available by the authors on request.

## Supplementary Materials

The following supporting information can be downloaded at: https://www.mdpi.com/article/10.3390/ijms262411775/s1.
