# Supplementary material for: Total Panax notoginseng Saponins Repair the Epidermal Barrier by Regulating a Multi-Pathway Network: Insights from an Integrative RHE Model and Multi-Omics Study
Source: Int J Mol Sci. 2025 Dec 5;26(24):11775. doi: 10.3390/ijms262411775 (PMC12733117; doi:10.3390/ijms262411775)
Supplement: Supplementary file 1 [file ijms-26-11775-s001.zip › Supplementary material S12.pdf]

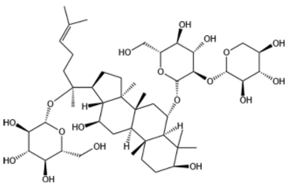

1. Notoginsenoside R1

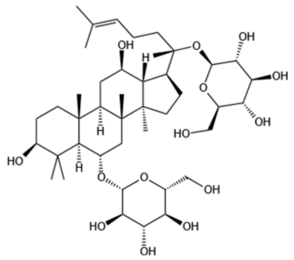

2. Ginsenoside Rg1

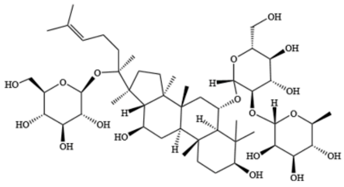

3. Ginsenoside Re

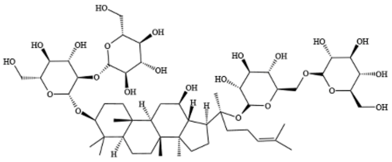

4. Ginsenoside Rb1

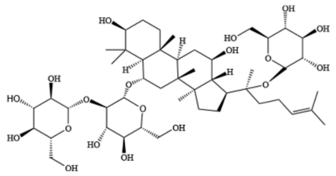

5. Ginsenoside Rd

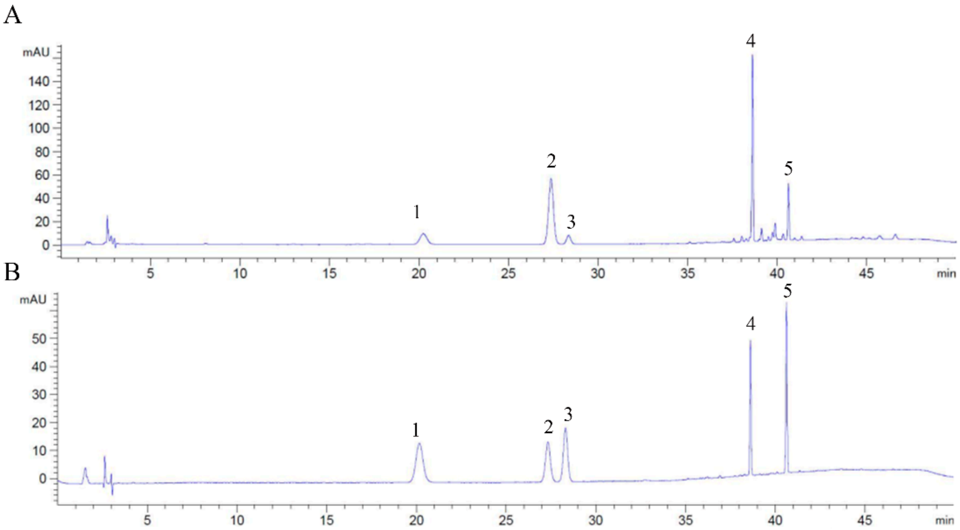

Figure S1. HPLC Chromatogram. **(A)** TSPN sample solution. **(B)** Mixed standard solution. **1**. Notoginsenoside R1. **2.** Ginsenoside Rg1. **3.** Ginsenoside Re. **4.** Ginsenoside Rb1. **5.** Ginsenoside Rd.

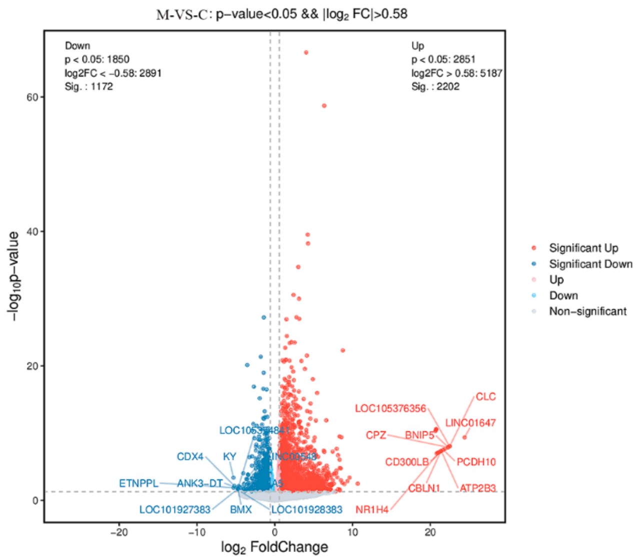

Figure S2. The volcano plot of DEGs between the model group and control group. .

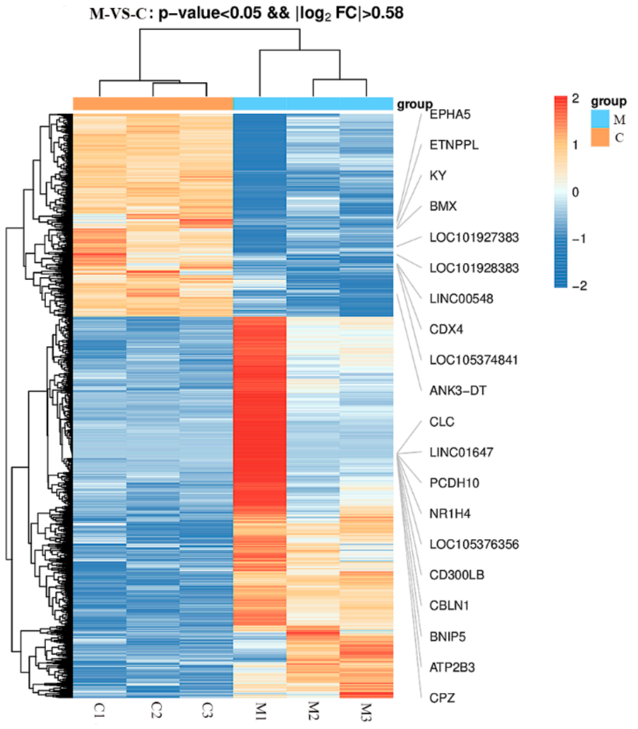

Figure S3. Heat map analysis showed the difference between the model group and control group in the expression of genes through the color of the block.

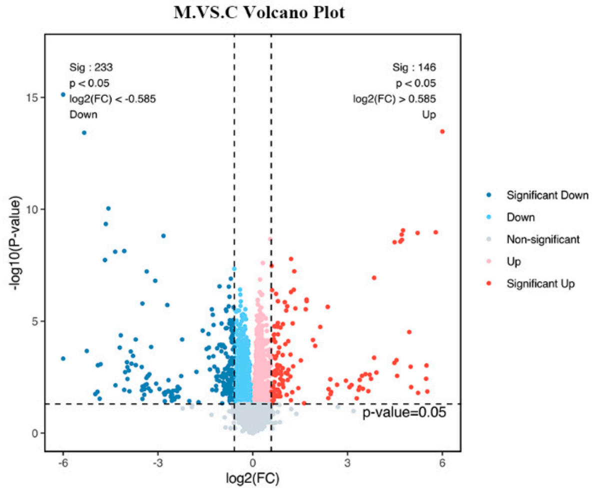

Figure S4. The volcano plot of DEPs between the model group and control group.

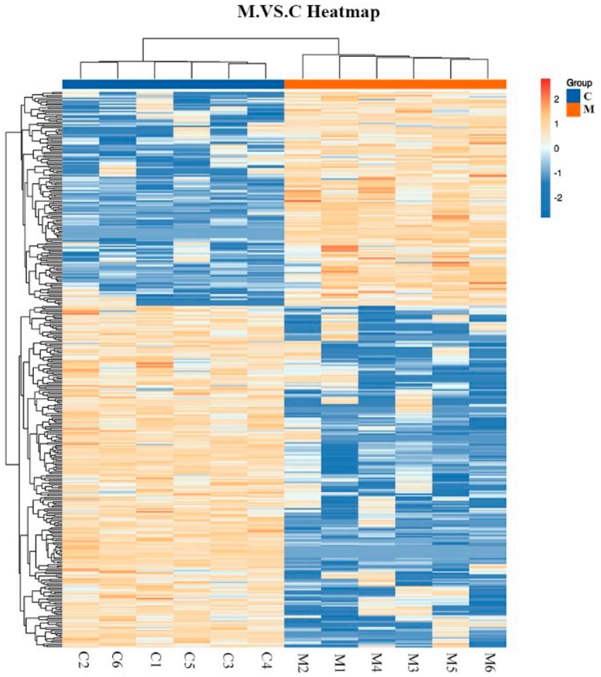

Figure S5. Heat map analysis showed the difference between the model group and control group in the expression of proteins through the color of the block.

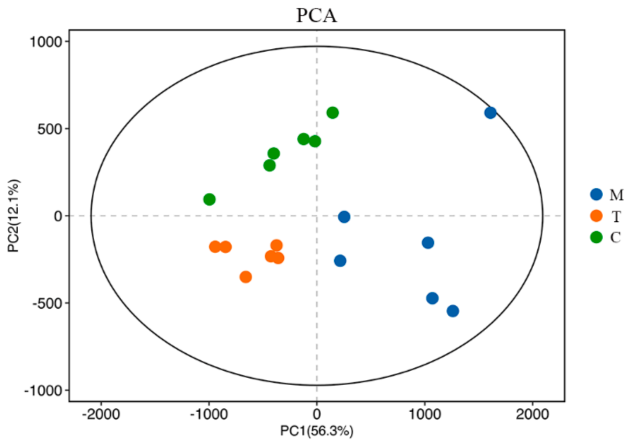

Figure S6. PCA score plots from Control, Model and TSPN group.

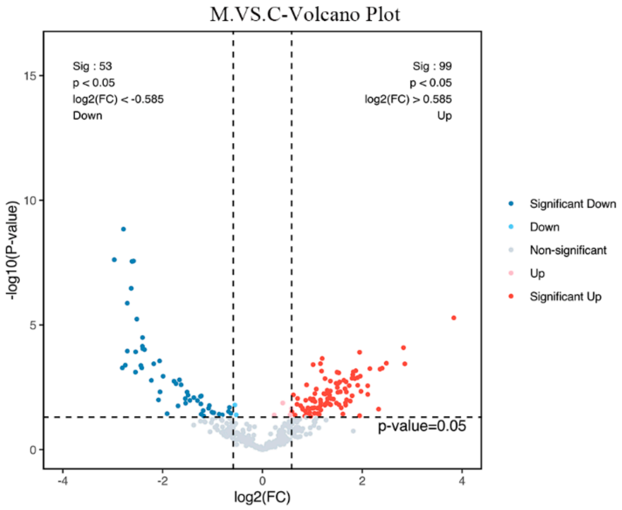

Figure S7. The volcano plot of DEMs between the model group and control group.

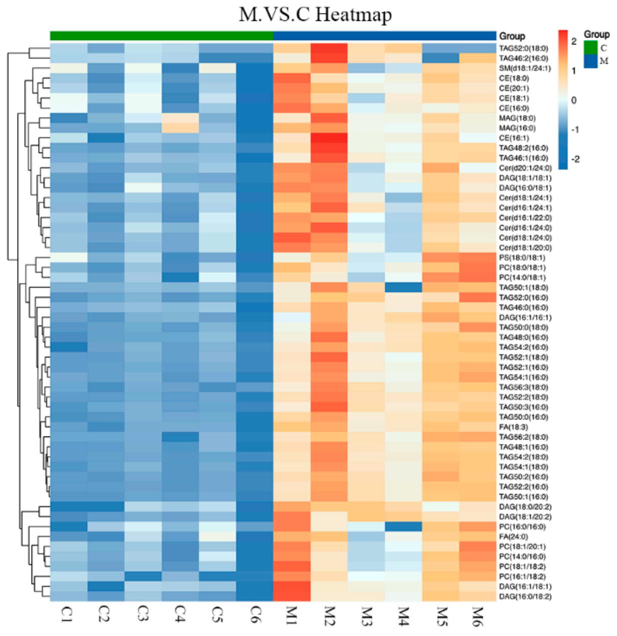

Figure S8. Heat map analysis showed the difference between the model group and TSPN group in the expression of lipid metabolites through the color of the block.

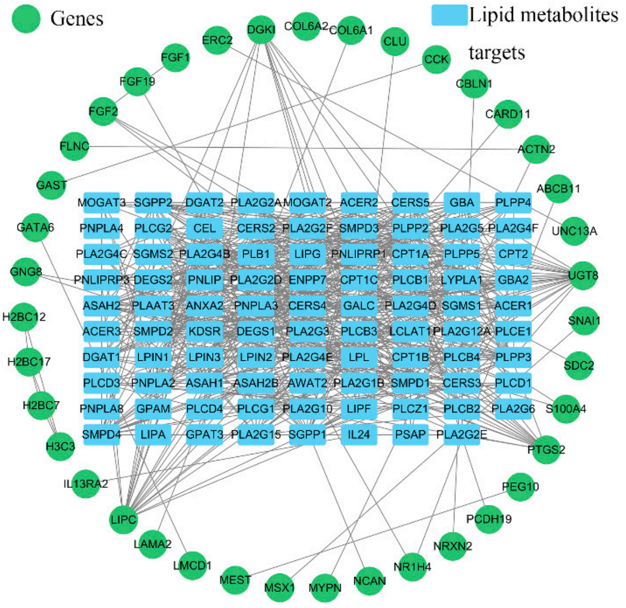

Figure S9. Transcription-lipid metabolite targets Interaction Network.
